# Participatory Environmentally Friendly Message Design: Influence of Message Features and User Characteristics

**DOI:** 10.3390/ijerph17041353

**Published:** 2020-02-20

**Authors:** Annie W. Y. Ng, Alan H. S. Chan

**Affiliations:** Department of Systems Engineering and Engineering Management, City University of Hong Kong, Kowloon Tong, Hong Kong, China; annieng.email@gmail.com

**Keywords:** environmental labels, sustainability, message design, participatory design

## Abstract

Labels and declarations are one of the tools of environmental management aimed at improving human behavior with regard to the environment. The development process of environmental labels and declarations has been strongly recommended to include participatory consultation with users. Research studies on participatory design of environmentally friendly messages, however, have never been seen. The purpose of this study was to examine participatory environmentally friendly message design with consideration for the effects of message characteristics and user factors. Forty Hong Kong Chinese people adopted a participatory draw-and-tell approach by drawing 26 environmentally friendly messages related to eco-products, energy conservation, and recycling and waste management, and then verbally described their design drawings. The results showed that environmentally friendly messages which were familiar, concrete, easy to visualize, and contained clear context were favored by users, and users with high object imagery preference benefited most from the participatory design through the draw-and-tell approach. This study fills the gap in the literature about participatory design in environmental labels and declarations which are used for promoting eco-friendly behavior in daily life. The findings should help facilitate the participatory development process of environmentally friendly messages for conveying pro-environmental actions.

## 1. Introduction

Given the increase in the impact that human activity has on the environment, people must now play an increasing role in working towards overall sustainability to raise their quality of life, especially in terms of their consumption patterns and pro-environmental behaviors. Labels and declarations are one of the tools of environmental management aimed at improving human behavior with regard to the environment, by providing information about the environmental aspects of products and services, including natural resource extraction, manufacture, distribution, use, and disposal [1]. These labels and declarations can be in the form of a statement, a symbol or a graphic on a product or package label, in product literature, in technical bulletins, in advertising, or in publicity tools [2]. Generally, pictorials were perceived as more effective than text statements in delivering warning messages [3].

BS EN ISO 14025 highly recommends that the process of development of environmental labels and declarations should be an open, participatory consultation with interested parties, such as purchasers, consumers, users, material suppliers, manufacturers, trade associations, non-governmental organizations, and public agencies [4]. ‘Participatory’ refers to the provision of opportunities for people to participate, and ‘consultation’ is the act of talking about something that is being decided with another person or a group of people in the form of a speech or piece of writing, which provides information, ideas, and opinions about something [5]. Recently, attention has been directed to the integration and involvement of users in the conceptual design process, also known as participatory design [6,7,8,9,10,11,12,13,14,15]. With user participation in the process of information design, the intended messages are more likely to be better conveyed and understood [16,17]. Baumeister and Onkila [18] identified five criteria that are essential for the development of eco-labels to support behavioral changes, namely creditability, comparability, clarity, transparency, and participation. Among these five criteria, stakeholder participation in the development of an eco-label appeared to be the most challenging to achieve. The participatory process is particularly important in empowering users as collaborators, deepening understanding of user experiences and perceptions as well as contributing to improved choices for pro-environmental interventions and stimulation of behavioral changes to produce long-term effects [19,20].

Previous studies on environmental labels and declarations focused mainly on topics such as consumer interpretation of recycling signs on packaging [21] and the influence of eco-labels on making sustainable purchasing choices [22]. Research studies on participatory design of environmentally friendly messages, however, have never been seen. The participatory design of infographics for public message representation would probably be based on daily experience and observation, learned knowledge and reasoning, personal characteristics of the users, and external reality characteristics [23,24]. The purpose of this study is to examine participatory environmentally friendly message design with consideration for the effects of message characteristics (concreteness, context availability, familiarity, and ease of visualization) and user factors (cognitive style, pro-environmental attitude, pro-environmental behavior, and gender). This research question to be addressed was “do message characteristics and user factors have significant effects on participatory environmentally friendly message design?”

The draw-and-tell approach was used to gather user experiences and perceptions towards the participatory environmentally friendly message design. Environmentally friendly messages were presented to the participants one by one. The participants drew the pictures based on their experiences and perceptions upon seeing the messages using the tablet provided to them. They were also asked to give a verbal description and to explain each of their drawings during or just after creating them. The draw-and-tell approach was adopted because, compared to either speech or writing alone, it provided a more thorough insight into user thoughts, especially with regard to the spatial relations and abstract notions of objects [25,26,27]. Angell et al. [28] reported that a draw-and-tell approach enables participation, in which users are given time and space to think, and facilitates the building of central ideas or key themes. Cleland and Wyborn [29] showed that drawings in participatory studies encourage people to demonstrate the details of the values, behaviors, and abstract concepts that they are projecting to the world. The preference toward participating in participatory environmentally friendly message design, degree of ease in drawing the intended message representations, and the degree of agreement with using a drawing method to illustrate the intended message representations were dependent variables of this study.

Environmental labels and declarations are a management tool which mainly provides environmental aspect information of the life cycle of products and services for improving human behavior with regard to the environment. Pro-environmental behaviors are related to emotional association with nature and not restricted to green purchasing; that is, they include saving water and energy as well as recycling waste [30,31,32]. Barr et al. [33] found that pro-environmental behaviors that could be performed in and around the home can be clustered in terms of purchase-oriented behaviors, habitual actions to conserve energy, and waste management practices. Therefore, in this research study, three categories of environmentally friendly messages covering common types of pro-environmental behavior, namely eco-product-related messages (e.g., energy-conserving electric products), energy conservation (e.g., turn off the lights), and recycling and waste management (e.g., no dumpling into sea or river), were studied. The findings of this study should help to fill the gap in the literature on participatory environmentally friendly message design and development. Moreover, the findings can aid stakeholders and practitioners to develop effective environmentally friendly messages that clearly convey appropriate pro-environmental actions to people in a particular context through a participatory design approach.

## 2. Method

### 2.1. Participants

Forty Chinese undergraduate students in Hong Kong (18 males and 22 females) took part in the study. The age range of the participants was 18 to 30 years (mean = 21.7 years, standard deviation = 1 year). Most of the participants (90%) were from engineering disciplines and the remaining participants studied business analysis and linguistics. Undergraduate students were recruited for the study because they are potential green purchasers and a target group for pro-environmental education. They are the lead to behave environmental friendly, adopt environmentally responsible attitudes, create a sustainable environment, and combat environmental problems in the future [34]. Their responses would provide the information necessary to develop effective environmentally friendly message through participatory design approach. This research was approved by the Research Committee of the City University of Hong Kong. Informed consent was obtained from each participant.

### 2.2. Environmentally Friendly Messages

Three categories of 26 environmentally friendly messages, namely, recycling and waste management, energy conservation, and eco-product-related messages, were used. They were identified with reference to common pro-environmental behaviors in daily life [1,2,4,21].

(a) Recycling and waste management (n = 9 where n is number of messages): please recycle unwanted copies, please flatten boxes, materials for compost, put all trash in sealed plastic bags, deposit recyclables here, no littering, owners must clean up after their dogs, reduce carbon dioxide, and no dumping into sea or river.
(b) Energy conservation (n = 6): turn off the lights, use natural light when possible, turn off computers after office hours, turn off the taps, keep the temperature at a moderate level, and use both sides of paper.
(c) Eco-product-related messages (n = 11): contains organically-grown ingredients, never tested on animals, free of petroleum by-products, no artificial fragrances and colorants, non-toxic material-based product, energy-conserving electric product, solar energy product, not genetically modified, energy-conserving car, eco fuel (e.g., unleaded gasoline), and energy-saving light bulb.

Each participant had a different randomized presentation sequence of the 26 environmentally friendly messages.

### 2.3. Apparatus and Instruments

The draw-and-tell research method was adopted to gather user experiences and perceptions towards the participatory environmentally friendly message design. The user factors and message characteristics were assessed by using Object–Spatial Imagery and Verbal Questionnaire (OSIVQ [35]), New Ecological Paradigm (NEP [36]) scale, Pro-environmental Behavior Measure (PEB [37]), and customized evaluation questionnaire.

The Object–Spatial Imagery and Verbal Questionnaire (OSIVQ) was used to assess object imagery preferences, spatial imagery preferences, and verbal cognitive styles of the participants [35]. The questionnaire consisted of 15 object imagery preference items (e.g., ‘I can close my eyes and easily picture a scene that I have experienced’), 15 spatial imagery preference items (e.g., ‘In school, I had no problems with geometry’), and 15 verbal cognitive style items (e.g., ‘I tell jokes and stories better than most people’). Each item was rated on a five-point scale with 1 = totally disagree and 5 = totally agree.

The New Ecological Paradigm (NEP) scale was used to measure the pro-environmental attitudes of the participants [36]. This scale consisted of 15 statements that address various aspects of an environmental worldview (e.g., ‘The earth is like a spaceship with very limited room and resources’ and ‘Humans are severely abusing the environment’). Each statement was rated on a five-point scale, with 1 being totally disagree to 5 being totally agree.

The Pro-environmental Behavior (PEB) Measure was used to measure the pro-environmental behaviors of the participants [37]. It consisted of 24 items, seven of which were about the last time the participants took pro-environmental actions (e.g., bought a product to save water, installed a renewable energy system at home). The items were assessed on a scale of never (0), 5 or more years ago (1), 1–3 years ago (2), and last year (3). The remaining 17 items were about the frequency of taking pro-environmental actions (e.g., turn off lights you are not using, save water by taking short showers); they were rated on a scale of never (0), occasionally (1), often (2), and always (3).

The customized evaluation questionnaire was designed for the participants to rate the four characteristics for each of the 26 environmentally friendly messages (concreteness, context availability, ease of visualization, and familiarity) used in this study. Concrete messages denote an actual substance or thing, whereas abstract messages represent something apart from real material or object basis. High context availability indicates the situation where people can easily associate a message with a certain circumstance in which the message would appear; otherwise, the message is said to be low context availability. Ease of visualization refers to how people can easily construct a visual mental picture of a message, and familiarity refers to the frequency with which an environmentally friendly message has been seen previously in daily life. On each page of the questionnaire, an environmentally friendly message and four boxes were provided for the participants to specify their 0–100 scores on perceived concreteness of the message (0 = very abstract, 100 = very concrete), context availability (0 = very difficult to determine, 100 = very easy to determine), ease of visualization (0 = very difficult to visualize, 100 = very ease to visualize), and familiarity with the message (0 = very unfamiliar, 100 = very familiar). Three closed-ended questions were also designed and placed at the end of the questionnaire to collect the preference toward participating in participatory environmentally friendly message design, degree of ease in drawing intended message representations, and degree of agreement with using a drawing method to illustrate intended message representations of the participants: (i) do you like participating in the task of environmentally friendly message design? (strongly dislike, dislike, neutral, like, strongly like); (ii) do you think it is easy to draw intended message representations (not very easy, not easy, neutral, easy, very easy); (iii) do you agree that drawing is a good method for illustrating your intended message representations? (strongly disagree, disagree, neutral, agree, strongly agree).

A touch-screen tablet (Samsung Galaxy, 10.1-inch monitor, dual core 1.6 GHz processor; manufactured by Samsung Electronics, South Korea) with S Note software and a stylus were provided to enable each participant to draw and complete questionnaires. An audio recorder was used to record the participants’ verbal descriptions and explanations about their drawings.

### 2.4. Procedure

Participants were informed that they were asked to participate in environmentally friendly message design through the draw-and-tell approach. Environmentally friendly messages were presented to the participants one by one. The participants drew the pictures upon seeing the messages using the tablet provided to them. Ten different colors (red, orange, yellow, green, blue, white, black, purple, brown, and gray) were available and the participants picked the colors that they favored during drawing. The participants were asked to draw each picture as simply as possible and as large as the space allowed, and to avoid using numerals and words in their drawings. They were also asked to give a verbal description and to explain each of their drawings during or just after creating them. After completing each drawing and description, participants rated their perceived context availability of, familiarity with, concreteness of, and ease of visualizing the environmentally friendly messages on the customized evaluation questionnaire.

At the end of the study, the participants answered the three closed-ended questions about their preference toward participating in environmentally friendly message design, the degree of ease in drawing intended message representations, and the degree of agreement with using a drawing method to illustrate intended message representations. They were also asked to complete the OSIVQ, NEP, and PEB. In the OSIVQ test, the participants rated each of the 45 items relating to object imagery preference, spatial imagery preference, or verbal cognitive style. For the NEP scale, the participants assessed each of the 15 items relating to their own environmental worldview. For the PEB measure, the participants determined their seven recent pro-environmental actions and the frequencies of taking each of the 17 pro-environmental actions. Each participant took approximately 90 min to finish the entire study.

## 3. Results

Altogether, 1040 drawings of the environmentally friendly messages were created by the participants. Three judges were invited to assess the drawings and corresponding transcribed verbal descriptions independently. For each message, the judges sorted the drawings into groups according to those that used very similar types of pictorial elements for environmentally friendly messages. They then compared the verbal descriptions with the drawings to further understand the ideas that the participants tried to convey in the drawings. Then the judges met and discussed their sorting results and, after due consideration, arrived at agreed reconciliations with regard to their independent assessments.

**Figure 1:**
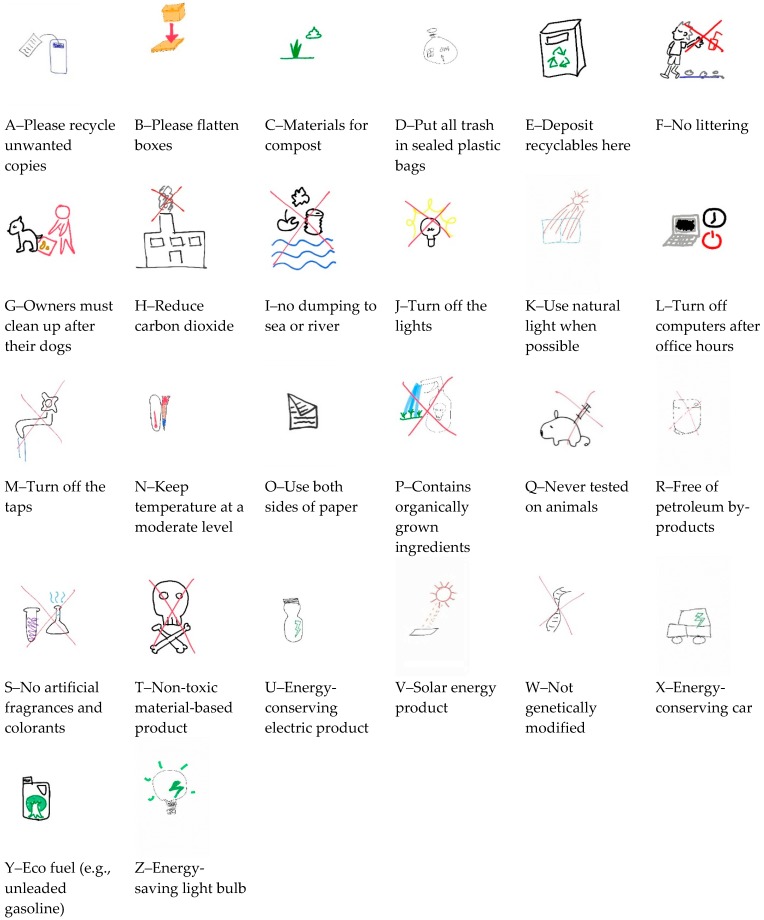
Samples of common representations for each environmentally friendly message from users.

Table 1 shows the common pictorial representation contents, number of representation ideas, and strength of stereotype for each environmentally friendly message. Samples of the most frequently drawn pictures for each environmentally friendly message are shown in Figure 1. The participants developed a variety of representative ideas for the environmentally friendly messages. The drawings that contained the same pictorial elements were recognized as having similar representation ideas for an environmentally friendly message. The number of representation ideas per environmentally friendly message varied from 6 (I–no dumping to sea or river) to 25 (U–energy-conserving electric product), with a mean of 16.04 and standard deviation of 5.20. Strength of stereotype indicated the degree of agreement among users relating to the most common interpretation of an environmentally friendly message, and was evaluated in terms of the proportion of common responses to total responses for a message. Stereotype strength per environmentally friendly message ranged from 12.50% (L–turn off computers after office hours) to 70.00% (T–non-toxic material-based product), with a mean of 28.70% and standard deviation of 12.52%. The number of representation ideas was negatively related to the strength of the stereotype (r_s_ = −0.808, n = 26, *p* < 0.01).

The mean ratings on preference toward participating in environmentally friendly message design (3.43) and the agreement with using a drawing method to illustrate intended message representations (3.80) exceeded the midpoint (three of the five-point rating scale), whereas the mean rating on the ease in drawing the intended message representations was 2.78. Therefore, the participants generally had a preference toward participating in environmentally friendly message design, but did not perceive the activity of drawing their intended message representations as easy. The preference toward participating in environmentally friendly message design was significantly associated with the degree of ease in drawing intended message representations (r_s_ = 0.507, n = 40, *p* = 0.001) and degree of agreement with using a drawing method to illustrate intended message representations (r_s_ = 0.640, n = 40, *p* < 0.001). The degree of ease in drawing intended message representations was significantly correlated with the degree of agreement with using a drawing method to illustrate intended message representations (r_s_ = 0.696, n = 40, *p* < 0.001).

### 3.1. Message Characteristics

The mean ratings for the four message characteristics of familiarity, context availability, ease of visualization, and concreteness were 57.13, 60.08, 65.25, and 75.72, respectively, all of which exceeded the midpoint (50) in the 0–100 rating scale. The concreteness, context availability, ease of visualization, and familiarity for each message are shown in Table 1. Message F (no littering) received the highest rating for all four characteristics, i.e., context availability, 86.38; familiarity, 91.13; ease of visualization, 89.50; and concreteness, 88.75. In contrast, message R (free of petroleum by-products) received the lowest rating for all the four characteristics, i.e., context availability, 31.25; familiarity, 27.38; ease of visualization, 34.63; and concreteness (46.00). Spearman correlation analysis revealed that the four message characteristics were associated with one another (see Table 2).

Correlation analysis also showed that strength of stereotype was positively related to ease of visualization and concreteness. The number of representation ideas was negatively correlated with concreteness, ease of visualization, and familiarity. Concreteness was significantly correlated with the preference toward participating in environmentally friendly message design. The degree of ease in drawing intended message representations was significantly related to concreteness and ease of visualization. The extent of agreement with using a drawing method to illustrate intended message representations was significantly correlated with concreteness, ease of visualization, and context availability.

### 3.2. User Factors

The user factors of gender, object imagery preference, spatial imagery preference, verbal cognitive style, New Ecological Paradigm (NEP), and Pro-environmental Behavior (PEB) were examined. The mean scores on Object–Spatial Imagery and Verbal Questionnaire (OSIVQ), i.e., object imagery preference, 3.29; spatial imagery preference, 3.19; and verbal cognitive style, 3.11, exceeded the midpoint (three of the five-point rating scale), thereby suggesting that in general the participants had a preference toward object imagery, spatial imagery, and verbal processing. The NEP scores of the participants ranged from 43 to 66 (mean = 51.2) (total score of 75), and the PEB scores ranged from 15 to 35 (mean = 23.45) (total score of 72). This result indicated that their pro-environmental attitude was high and that their pro-environmental behavior was low.

The Mann–Whitney U test showed that gender exerted a significant effect on the degree of ease in drawing intended message representations (χ^2^ = 4.057, df = 1, *p* = 0.044; male = 3.11 and female = 2.50) and degree of agreement with using a drawing method to illustrate intended message representations (χ^2^ = 5.928, df = 1, *p* = 0.015; male = 4.17 and female = 3.50). The ratings of the males on the ease of drawing intended message representations and agreement with using a drawing method to illustrate intended message representations were significantly higher than those of the females. Correlation analysis showed that the degree of ease in drawing intended message representations was significantly related to verbal cognitive style (r_s_ = −0.313, n = 40, *p* = 0.049) and object imagery preference (r_s_ = 0.420, n = 40, *p* = 0.007). The degree of agreement with using a drawing method to illustrate intended message representations was significantly associated with verbal cognitive style (r_s_ = −0.316, n = 40, *p* = 0.047). No other significant effects were found.

## 4. Discussion

The purpose of this study was to investigate the effects of user factors and message characteristics on participatory environmentally friendly message design using the draw-and-tell approach. The drawings and their corresponding verbal descriptions indicated the expression of users towards environmentally friendly messages.

### 4.1. Message Characteristics

**Figure 2:**
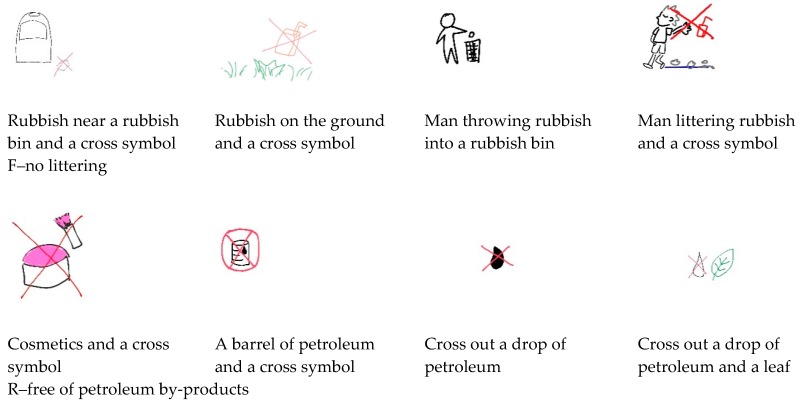
Examples of representation for the least and most familiar, concrete, and easily visualized environmentally friendly message.

Familiarity, concreteness, and ease of visualization exerted significant effects on environmentally friendly message representations. The representation ideas used for environmentally friendly messages were less varied when the messages were familiar, concrete, and easy to visualize. For instance, for the most familiar, concrete, and easily visualized environmentally friendly message (F–no littering) in this study, the 40 participants proposed eight different ideas, such as ‘rubbish near a rubbish bin and a cross symbol,’ ‘rubbish on the ground and a cross symbol,’, ‘man throwing rubbish into a rubbish bin’, and ‘man littering rubbish and a cross symbol.’ For the least familiar, least concrete, and least easily visualized environmentally friendly message (R–free of petroleum by-products), 16 various representations were proposed; examples included ‘cosmetics and a cross symbol,’ ‘a barrel of petroleum and a cross symbol,’ ‘cross out a drop of petroleum’, and ‘cross out a drop of petroleum and a leaf.’ Examples of representation for the least and most familiar, concrete, and easily visualized environmentally friendly message are shown in Figure 2. These results support the previous findings of Yilmaz et al. [38] which indicated that a person’s previous experience plays an important role in their ability to generate solutions to a problem; familiarity with a problem does not necessarily yield a large solution space, and a prototypical solution is usually available for a familiar problem. Gonalves et al. [39] also found a relationship between the abstractness of textual stimuli and the number of representation ideas produced by their participants. The number of ideas for stimuli generally improves from extremely concrete textual stimuli to abstract ones; however, the number of ideas seems to decrease when the abstraction of a stimulus reaches a certain level.

An understanding of the population stereotypes of users regarding environmentally friendly messages should help in the development of effective user-oriented environmentally friendly messages. This study found that strength of stereotype was significantly related to ease of visualization and concreteness of environmentally friendly messages. When the concreteness and ease of visualization of environmentally friendly messages were high, the agreement among user-intended message representations for the messages was also high. For instance, message T (non-toxic material-based product), which had the highest stereotype strength (70.00%), also received high concreteness rating (82.25%) and ease of visualization rating (77.25%); the most common intended message representation related to this message was ‘skull and a cross symbol’. To enhance the chance of obtaining commonly shared intended message representations of environmentally friendly messages from users, the messages must be easy to visualize and be concrete enough for the users to relate to. Environmentally friendly messages that are considered less concrete and less easy to visualize should be treated with caution during the participatory design process, for instance, by providing supplementary background information about the messages to help participants express their intended message representations.

All three preference measures were dependent on message concreteness. The preferences toward participatory environmentally friendly message design, perceived ease in drawing intended message representations, and agreement with using a drawing method to illustrate intended message representations were high for concrete messages. A previous study by Wojtowicz and Butelski [40] showed that ease of visualization at the early design stage enabled users to develop a fuller understanding of design proposals from concepts. In the present study, the results indicated that when an environmentally friendly message was easy to visualize, users could more easily draw intended message representations and showed a high degree of agreement with using a drawing approach. The context in which environmentally friendly messages appear, i.e., context availability, was significantly and positively associated with perceived agreement with using a drawing method to illustrate intended message representations. Overall, the research findings showed that environmentally friendly messages that were concrete, were easy to visualize, and contained clear context were definitely favored by users for representing messages pictorially.

### 4.2. User Characteristics

Among the cognitive styles used, object imagery preference was significantly associated with perceived ease in drawing intended message representations. The higher the object imagery preference, the easier it was to draw the environmentally friendly message representation. Participants with high object imagery preference were good in drawing their intended environmentally friendly message representations. A recent study by Kibar and Akkoyunlu [41] revealed that object–visual individuals use an effective and holistic approach to define the general view of object characteristics. Object visualizers use imagery to construct vivid, colorful, pictorial, and detailed images of objects [41,42]; create images of objects and process visual information as a whole [43]; and process the visual appearance of objects and scenes in terms of their resemblance to their shape, size, color, and texture information [35,36]. These characteristics of object visualizers explain why participants in this study with a strong object imagery preference perceived ease in drawing their intended representations of environmentally friendly messages. These results imply that people with these characteristics will benefit from participatory environmentally friendly message design through drawing.

Verbal cognitive style was significantly but negatively associated with perceived agreement with using a drawing method to illustrate message representations and perceived ease in drawing intended message representations. The higher the verbal preference was, the lower the agreement with using a drawing method to illustrate environmentally friendly message representations and the lower the perceived ease in drawing intended message representations. Individuals with a strong verbal cognitive style have been shown to prefer using verbal techniques to process information [44], verbal thinking i.e., using words to think [45], and verbal–analytical strategies [41]. It has also been reported that individuals with a strong verbal cognitive style are effective in verbal tasks [41], draw a few items, and present few details in human figure drawing tests [46]. Therefore, for people with high verbal cognitive preference, solely using a drawing method would not be suitable for them to produce intended representations of environmentally friendly messages.

Compared to the females, the males significantly perceived that drawing intended representations of environmentally friendly messages was easy and agreed with the use of a drawing method to illustrate environmentally friendly message representations. The results indicated the need to further improve the drawing approach to help users, especially females, in generating their intended representations of environmentally friendly messages. Regarding the user characteristics of pro-environmental attitude and behavior, the results of this study indicated that users with different environmental attitudes and behaviors did not differ in providing valuable suggestions for environmentally friendly messages.

Caution should be exercised in generalizing the outcomes of this study to non-Chinese people because all the participants in this study were Chinese with largely Chinese cultural backgrounds.

## 5. Conclusions

Research studies on participatory design of environmentally friendly messages are limited. The purpose of this study was to examine participatory environmentally friendly message design with consideration the effects of message characteristics and user factors. The findings fill a gap in the literature about the participatory environmental labels and declarations design. This study revealed the population stereotypes on environmentally friendly messages related to eco-products, energy conservation, and recycling and waste management. A better understanding of population stereotypes about environmentally friendly messages would help develop effective user-centered environmentally friendly messages for the general population to take appropriate environment protection and sustainability actions. With consideration of the message characteristics effect on participatory environmentally friendly messages design, to create commonly shared intended representations of environmentally friendly messages, the messages must be easy to visualize, be concrete, and be familiar to users. These message characteristics also had an effect on preferences toward participatory environmentally friendly message design and the drawing approach for illustrating intended message representations. Environmentally friendly messages that do not satisfy these characteristics should be given very careful consideration during the participatory development process, for example, by provision of supplementary background information about the messages to help the users to express their intended representations during the participatory process. Regarding to the influence of user factors, it was found that pro-environmental attitude and behavior did not have any significant effect on the participatory environmentally friendly message design. People with different pro-environmental attitude and behavior can be asked to contribute in participatory design. Individuals with high object imagery preference also benefitted the participatory environmentally friendly message design. For those with verbal cognitive preference, there is a need to further improve the participatory draw-and-tell approach to facilitate them in illustrating intended representations of environmentally friendly messages. The findings of this study can facilitate practitioners to use the participatory design approach in the future to develop more practically useful environmentally friendly messages for effectively transmitting pro-environmental actions to the public so as to improve the quality of life.

## Figures and Tables

**Table 1:** Number of representation ideas, most common representations, strength of stereotype, concreteness, context availability, ease of visualization, and familiarity for the environmentally friendly messages.

	Environmentally Friendly Message	Number of Representation Ideas	Stereotype Strength (%)	Concreteness	Context Availability	Ease of Visualization	Familiarity
A	Please recycle unwanted copies	8	32.50	88.50	73.50	80.50	76.63
B	Please flatten boxes	14	32.50	81.00	38.63	67.50	36.38
C	Materials for compost	18	20.00	61.25	35.38	46.63	33.38
D	Put all trash in sealed plastic bags	10	37.50	85.25	51.75	73.38	48.25
E	Deposit recyclables here	15	20.00	81.25	74.88	74.13	73.13
F	No littering	8	40.00	88.75	86.38	89.50	91.13
G	Owners must clean up after their dogs	15	30.00	85.88	78.88	75.75	77.38
H	Reduce carbon dioxide	22	22.50	75.00	50.75	60.63	59.50
I	No dumping to sea or river	6	35.00	85.00	75.75	77.88	71.75
J	Turn off the lights	20	22.50	88.25	68.38	73.00	64.00
K	Use natural light when possible	19	15.00	79.00	43.88	63.88	34.38
L	Turn off computers after office hours	22	12.50	79.38	68.50	64.88	56.75
M	Turn off the taps	13	45.00	86.50	79.38	78.50	71.50
N	Keep temperature at a moderate level	22	20.00	72.50	68.75	62.50	66.13
O	Use both sides of paper	10	35.00	86.75	64.75	80.13	74.00
P	Contains organically grown ingredients	19	20.00	65.38	59.00	60.88	55.50
Q	Never tested on animals	16	27.50	71.63	39.50	57.88	36.13
R	Free of petroleum by-products	16	27.50	46.00	31.25	34.63	27.38
S	No artificial fragrances and colorants	23	15.00	68.50	44.50	45.88	40.38
T	Non-toxic material-based product	8	70.00	82.25	70.75	77.25	70.25
U	Energy-conserving electric product	25	15.00	71.25	65.00	59.50	58.13
V	Solar energy product	17	20.00	73.63	66.00	66.50	65.38
W	Not genetically modified	17	45.00	73.50	51.88	60.38	52.25
X	Energy-conserving car	16	27.50	64.00	59.13	56.63	45.00
Y	Eco fuel (e.g., unleaded gasoline)	20	25.00	51.88	40.50	41.00	31.88
Z	Energy-saving light bulb	18	20.00	76.38	75.00	67.25	68.88
	Minimum	6	12.50	46.00	31.25	34.63	27.38
	Maximum	25	70.00	88.75	86.38	89.50	91.13
	Mean	16.04	28.17	75.72	60.08	65.25	57.13
	Standard deviation	5.20	12.52	11.22	15.66	13.16	17.24

**Table 2:** Correlation coefficients among message characteristics, number of representation ideas, strength of stereotype, preference toward participating in participatory environmentally friendly message design, degree of ease in drawing intended message representations, and degree of agreement with using a drawing method to illustrate intended message representations.

	Familiarity	Concreteness	Ease of Visualization	Context Availability
Familiarity	1			
Concreteness	0.768 **	1		
Ease of visualization	0.849 **	0.9947 **	1	
Context availability	0.908 **	0.669 **	0.756 **	1
Number of representation ideas	−0.467 *	−0.593 **	−0.700 **	−0.369
Stereotype strength	0.327	0.487 *	0.506 **	0.236
Preference toward participating in participatory design	0.067	0.317 *	0.074	0.158
Degree of ease in drawing	0.051	0.454 **	0.561 **	0.171
Degree of agreement with using a drawing method	0.293	0.411 **	0.375*	0.359 *

** Correlation is significant at the 0.01 level (2-tailed), * Correlation is significant at the 0.05 level (2-tailed).
